# Oxygen and Hydrogen Stable Isotope Ratios of Bulk Needles Reveal the Geographic Origin of Norway Spruce in the European Alps

**DOI:** 10.1371/journal.pone.0118941

**Published:** 2015-03-05

**Authors:** Yuri Gori, Ron Wehrens, Nicola La Porta, Federica Camin

**Affiliations:** Research and Innovation Centre, Edmund Mach Foundation (FEM), Via E. Mach 1, 38010 San Michele all’ Adige, Trento, Italy; University of Western Sydney, AUSTRALIA

## Abstract

**Background:**

Tracking timber is necessary in order to prevent illegal logging and protect local timber production, but there is as yet no suitable analytical traceability method. Stable isotope ratios in plants are known to reflect geographical variations. In this study we analysed four stable isotope ratios in order to develop a model able to identify the geographic origin of Norway spruce in the European Alps.

**Methodology and Principal Findings:**

δ^18^O, δ^2^H, δ^13^C and δ^15^N were measured in bulk needles of *Picea abies* sampled in 20 sites in and around the European Alps. Environmental and spatial variables were found to be related to the measured isotope ratios. An ordinary least squares regression was used to identify the most important factor in stable isotope variability in bulk needles. Spatial autocorrelation was tested for all isotope ratios by means of Moran’s *I*. δ^18^O, δ^2^H and δ^15^N values differed significantly between sites. Distance from the coast had the greatest influence on δ^2^H, while latitude and longitude were strongly related to δ^18^O. δ^13^C values did not appear to have any relationship with geographical position, while δ^15^N values were influenced by distance from the motorway. The regression model improved the explanatory power of the spatial and environmental variables. Positive spatial autocorrelations were found for δ^18^O and δ^2^H values.

**Conclusions:**

The δ ^18^O, δ^2^H and δ^15^N values in *P*. *abies* bulk needles are a suitable proxy to identify geographic origin as they vary according to geographical position. Although the regression model showed the explanatory variables to have significant power and stability, we conclude that our model might be improved by multivariate spatial interpolation of the δ ^18^O and δ^2^H values.

## Introduction

Illegal logging practices are thought to be the major cause of worldwide deforestation [1], [2], which is responsible for the loss of natural resources and global biodiversity and for up to 30% of human-caused CO_2_ emissions. They therefore also play a key role in climate change. Moreover, illegal logging is associated with loss of biodiversity, the introduction and spread of harmful pests, and negative social and economic effects such as violation of ownership, corruption and tax evasion [2]. Illegal logging is widespread in non-tropical regions such as Albania and Georgia, as well as in tropical regions [3], [4], where it accounts for between 85% and 95% of global timber production with worldwide economic losses estimated at around US$ 10 billion per annum [5], [3]. A suitable method for combating illegal logging is therefore needed to assure consumers that the timber they purchase originates from sustainable managed forests. Some authors [6–9] have proposed DNA analysis as a tool for determining tree provenances. However, given that DNA fingerprints do not directly reflect geographic variation but rather variations in natural populations within the native area of a species, we argue that this method cannot be applied to plantation trees [10], [11].

Norway spruce (*Picea abies* Karst.) is a European species with an extensive natural range throughout Europe. Spruce stands account for almost all the forests in the Alps and they are hugely important to the mountain economy [12]. In recent decades the area under forest certification has greatly expanded in Europe [13], but although certification systems support timber traceability, there is to date no reliable analytical method to verify timber provenance [14].

Stable isotope ratios are known to reflect geographical variation and have therefore been widely used to trace the provenance of various agricultural products, such as meat [15], [16], cheese [17], wine, orange juice [18] and even drugs [19]. Carbon isotope ratios in plants are controlled by the ratio of intercellular to ambient CO_2_ concentrations which are in turn influenced by environmental factors such as irradiance, temperature, relative humidity and soil water availability [20–22]. Hydrogen and oxygen stable isotopes in plants reflect the isotopic composition of soil water and transpiration enrichment, which is mainly controlled by the stomatal opening [23], [24]. The isotopic composition of soil water is highly dependent on the isotopic composition of meteoric precipitation, which in turn is highly dependent on geographical location [23]. In addition, as previous studies have demonstrated [25], [26], distance from motorway and NO_2_ concentrations due to anthropogenic emissions are thought to influence δ^15^N in plant materials.

Some initial results from attempts to identify the geographical origin of wood using tree-ring δ^13^C in the south-western United States have been reported by Kagawa and Leavitt [10]. Horacek et al. [27] used the δ^13^C and δ^18^O values of bulk wood to discriminate between Siberian larch and European larch, while a study by Keppler et al. [28] found the δ^2^H values of the lignin methoxyl groups to correlate strongly with the OIPC (Online Isotope Precipitation Calculator; accessible at http://waterisotopes.org/) δ^2^H values of meteoric water. Despite these recent preliminary studies, a comprehensive method for identifying the geographic origin of forest trees has not yet been found.

Given that plant isotope ratios are mainly controlled by predictable geographically-dependent variables (e.g. environment and climate), we explored the relationship between four stable isotope ratios (δ^2^H, δ^18^O, δ^15^N and δ^13^C) in Norway spruce needles (*Picea abies* (L.) Karst) and these geographically-dependent variables in order to develop a predictive plant isotope model that could be used to estimate the geographic origin of trees. The novelty of our study lies mainly in the power of combining a multi-isotope analysis with eleven different explanatory variables which may have a direct physiological influence on isotope discrimination. Significant correlations were found between δ^13^C of needle bulk material and tree rings in *Picea abies* [29] and *Pinus sylvestris* [30]. Other studies have reported similar results for average wood and needle δ^2^H values over an 8-year study period [31]. Indeed wood and needle δ^2^H values are thought to reflect similar sources of climate information [32], and spruce needles are thought to best reflect the δ^15^N signal of NO_2_ [26] anthropogenic emissions. For these reasons, we chose *P*. *abies* bulk needle as plant materials for our stable isotope measurements.

## Methods

### Ethics statement

Our field studies did not involve endangered or protected species, the coordinates of the study locations are provided in Table 1.We sampled about 100–150 current-year needles of 100 *Picea abies* around European Alps and no animals were involved in this study. Sampling was only conducted in alpine public forests where no permits for were required.

**Table 1:** Sampled sites and related mean stable isotope values.

Site	Site code	δ^18^O	δ^2^H	δ^13^C	δ^15^N	Longitude	Latitude	Altitude (m)
Saint-Chaffrey	1	32.1 ± 0.76	-105 ± 3.38	-28.5 ± 0.82	-0.8 ± 0.44	6°59′	44°94′	1452
Villar-Perosa	2	29.2 ± 0.96	-130 ± 2.79	-27.7 ± 0.96	-3.6 ± 1.70	7°25′	44°92′	513
Cogne	3	28.2 ± 0.29	-139 ± 3.89	-27.9 ± 0.86	-4.8 ± 0.42	7°31′	45°64′	1378
Ayes	4	27.9 ± 0.16	-129 ± 2.41	-27.1 ± 0.48	-3.6 ± 0.19	7°73′	45°84′	1660
Medel	5	26.7 ± 0.36	-133 ± 2.10	-27.6 ± 0.92	-3.8 ± 0.98	8°85′	46°64′	1488
Mezzoldo	6	29.6 ± 0.26	-117 ± 4.08	-28.5 ± 0.57	1.0 ± 0.09	9°57′	46°13′	373
Savognin	7	27.3 ± 0.47	-141 ± 4.06	-28.1 ± 1.98	-0.9 ± 0.80	9°62′	46°58′	1306
Arnoga	8	28.0 ± 0.52	-120 ± 2.43	-27.5 ± 0.68	-6.2 ± 0.66	10°25′	46°46′	1830
Pfunds	9	28.9 ± 0.53	-142 ± 4.79	-27.6 ± 0.64	-2.1 ± 0.28	10°56′	46°97′	1141
Pellizzano	10	27.9 ± 0.42	-127 ± 3.46	-28.7 ± 0.30	-2.5 ± 0.27	10°76′	46°31′	986
Martell	11	27.8 ± 0.91	-129 ± 3.71	-27.8 ± 0.44	-1.5 ± 2.25	10°78′	46°55′	1360
Sautens	12	26.8 ± 0.37	-136 ± 3.44	-28.3 ± 0.97	-2.1 ± 0.29	10°89′	47°2′	801
Caldonazzo	13	29.0 ± 1.09	-108 ± 1.58	-29.0 ± 1.40	-7.4 ± 10	11°22′	46°05′	635
Primiero	14	27.1 ± 0.12	-113 ± 4.99	-28.1 ± 0.88	-0.5 ± 0.74	11°84′	46°18′	801
Agordo	15	25.4 ± 0.40	-111 ± 3.16	-28.5 ± 0.77	-1.2 ± 0.13	11°98′	46°25′	1112
Cortina	16	27.1 ± 0.47	-120 ± 1.41	-27.5 ± 0.54	0.2 ± 0.32	12°14′	46°6′	1493
Erto	17	27.1 ± 0.83	-104 ± 2.12	-29.1 ± 1.92	-3.1 ± 0.77	12°42′	46°29′	787
Cadore	18	26.5 ± 0.24	-117 ± 2.16	-27.3 ± 1.73	-5.8 ± 0.38	12°5′	46°46′	1174
Barcis	19	26.3 ± 0.64	-109 ± 2.63	-27.1 ± 0.64	-2.2 ± 0.35	12°55′	46°19′	433
Ugovizza	20	26.4 ± 0.70	-126 ± 2.86	-28.3 ± 0.53	-7.2 ± 0.20	13°47′	46°52′	1018

Pooled standard deviations were:

0.52 for δ^18^O; 3.07 for δ^2^H; 0.90 for δ^13^C; 0.61 for δ^15^N

**Fig 1:**
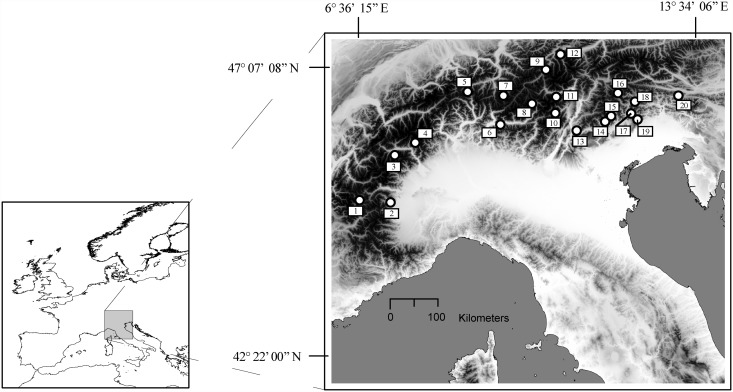
Location of the 20 sampling sites in and around the European Alps.

Longitude and latitude were determined by GPS. Mean values and standard deviations are reported for each stable isotope value (N = 5). Site codes are as reported in Fig. 1.

### Field sites

Twenty random locations in the Alps were generated using the random module in GRASS-GIS. The exact sites were those plots of Norway spruce (Corine Land Cover, version 16, April 2012; http://www.eea.europa.eu/) in closest proximity to these twenty points (Fig. 1). Each site covered an area of approx. 2000 m^2^ with elevations ranging from 373 to 1830 m (GPS GeoXT 3000 series, Trimble, Sunnyvale, USA). Five trees were selected in each plot and in December 2012 samples of current-year needles (n>100) were collected from each of these trees (irrespective of aspect and position in the crown). The stands consisted mainly of mature *P*. *abies*, but other woody species (*Abies alba* Miller, *Acer pseudoplatanus* L., *Fagus sylvatica* L. and *Larix decidua* Miller) were present at low densities [33]. Overall, the stands were dense with regular, continuous canopies and heterogeneous structures.

The local climate at the 20 sites was primarily dependent on altitude then on latitude, and ranged from sub-Mediterranean in the southern part to continental in the inner and northern parts.

### Explanatory variables

In order to reduce the risks associated with multiple comparisons [34] all the explanatory variables were chosen to represent environmental variables of hypothetical importance for stable isotope fractionation in trees [35], and a total of eleven explanatory variables were selected to develop a predictive plant isotope model.

### Climate variables: Mean annual temperature and autumn precipitation

As isotope fractionations of *P*. *abies* in the Alps may depend on autumn precipitation (21 September to 20 December) and mean summer and annual temperatures [36], we chose these proxies for our predictive model.

Temperature data were extracted using daily Land Surface Temperature (LST) maps (available from https://wist.echo.nasa.gov). Datasets were provided by the Terra and Aqua satellites, both of which carry MODIS sensors (Moderate Resolution Imaging Spectro-radiometer). MODIS-LST data have been collected since 2002 and provide very accurate daily minimum, maximum and mean temperatures in a raster georeferenced format with a high resolution of 200 m pixels. As MODIS-LST maps are affected by certain errors, such as cloud contamination and missing pixels, all daily temperature maps of the study area from January to December 2012 were processed using an algorithm developed by Neteler [37]. This algorithm allows very accurate temperature maps to be reconstructed in a GIS system (GRASS-GIS, version 7.0, available at http://grass.osgeo.org, GRASS Development Team, 2014) by filtering out clouds and poor quality pixels then filling the no-data areas in the maps using a temperature-gradient-based model [38].

Autumn precipitation was obtained from European Climate Assessment & Dataset maps (ECAD 5.0, available at: http://eca.knmi.nl/). ECAD precipitation maps were created by the EURO4M project (European Reanalysis and Observations for Monitoring) through interpolation of 2136 meteorological stations in Europe and the Middle East [39], [40]. All series were corrected and homogenised for long-term error to increase the strength of the climate signals [41]. Daily ECAD precipitation maps were imported using the *r.in.gdal* module and 2012 autumn precipitation was computed for the Alpine region using the *r.series* module; both algorithms were implemented in GRASS-GIS.

### Variables from Digital Elevation Model: Topographic wetness index and global irradiance

Soil features and soil water availability may affect isotope composition. As a proxy of soil moisture, we used the topographic wetness index (TWI), [42] which expresses the relative wetness of a site as its propensity to be saturated to the surface given the contributing area and local slope characteristics.

Solar radiation was quantified as an indicator of available energy for photosynthetic CO_2_ assimilation. Global irradiance, expressed in W/m^2^ for the whole annual period, and TWI were calculated using the European Digital Elevation Model (EU-DEM version 1, available at http://www.eea.europa.eu) at 25- m resolution using the *r.sun.daily* and *r.watershed* modules implemented in GRASS-GIS.

### Spatial variables

Besides environmental factors, altitude, distance from the coast and geographic location have a strong influence on plant isotopic composition [23], [43–45]. The coordinates (latitude and longitude expressed in degrees/minutes/seconds) and elevation in metres for each site were obtained with a GPS device (GeoXT 3000 series, Trimble, Sunnyvale, USA).

Moreover, distance from motorway and NO_2_ concentration due to anthropogenic emissions at each site have been reported [25], [26] to influence δ^15^N values in plant materials. In order to take into account these two variables as proxies for our predictive model, we extracted NO_2_ concentrations using European NO_2_ concentration maps (available from Interpolated Air Quality Dataset maps http://www.eea.europa.eu; spatial resolution was 10.000 meters), while the motorway shape was extracted using the specific tool available at: http://overpass-turbo.eu/.

Distances from the coast and from the motorway were computed using the *v*.*distance* module (GRASS-GIS).

### Isotope analysis

Needles were dried at 65°C for 72 h, then ground according to the method described by Laumer et al. [46]. Firstly, they were twice cooled (-196°C) in liquid nitrogen to convert them to a highly brittle state. In a second step, each cooled sample was placed in a stainless steel cylinder (25 ml) with a 10 mm grinding ball and pulverized by radial oscillation (1800 Hz min^-1^) for 4 minutes using a freezer-mill (CryoMill, Retsch, Hann, Germany). Liquid nitrogen circulated through the system to keep the temperature at -196°C. The procedure was repeated twice to obtain a final sample size of between 5 and 20 μm.

Samples of about 0.30±0.5 mg of the dried ground needles were placed in tin capsules for δ^13^C and δ^15^N analysis and in silver capsules for δ^18^O and δ^2^H analysis. All samples were then oven dried (80°C) to remove water vapour, then stored in a desiccator until analysis. The analysis was performed according to the procedure described in [36]. The values are expressed according to the IUPAC protocol[47]. The δ^2^H values were calculated against CBS (Caribou Hoof Standard δ^2^H = -197 ± 2 ‰) and KHS (Kudu Horn Standard, δ^2^H = -54 ± 1 ‰) through the creation of a linear equation [48]. We used these two keratinous standards because there is no international organic reference material with a similar matrix to ours. A sample of NBS-22, a fuel oil that doesn’t exchange, and one needle control sample were routinely included in each analytical run as a check of system performances. In both cases, we obtained highly repeatable results over the 2 month running period (standard deviation < 2 ‰).The samples analysed were normalised according to a single working standard calibrated against fuel oil NBS-22 (IAEA-International Atomic Energy Agency, Vienna, Austria) for δ^13^C and δ^15^N, IAEA-CH-6 sucrose for δ^13^C, and USGS 40 (U.S. Geological Survey, Reston, VA, USA) for both δ^13^C and δ^15^N. One control needle sample was routinely included in each analytical run as a check of system performances and we obtained highly repeatable results over the 2 month running period (standard deviation < 0.2 ‰). Around 20% of the samples were also analysed using the three international standards listed above and a calibration curve as suggested by IUPAC [47], resulting in a difference with the data normalised with a single standard always lower than 0.2‰.

Measurement uncertainty, expressed as 1 standard deviation when measuring the needle control sample 10 times, was 0.2 ‰ for δ^13^C and δ^15^N, 0.4 ‰ for δ^18^O and 2 ‰ for δ^2^H.

### Statistical analysis

Descriptive statistics (means, standard deviations) were carried out for all the stable isotope series and explanatory variables. In order to account for intra-site variability in the samples, we used a pooled standard deviation computed from the 5 samples from each site for all stable isotope values. Prior to analysis, data were examined graphically and tested for normality (Shapiro-Wilk test) and homogeneity of variance (Fligner-Killeen test for ANOVA).

The relationship between the stable isotope values and the explanatory variables was explored by Pearson’s correlation coefficient.

Multiple Ordinary Least Square Regression analyses (OLS) were carried out to predict the values of each stable isotope. A stepwise variable selection procedure was used to find the best set of explanatory variables. Starting with all the predictors in the model (11 explanatory variables), we used an F-test (*p* < 0.05) to identify the significant predictors which best accounted for the variation in stable isotope values.

The presence of spatial autocorrelation in isotope values was tested by means of Moran’s *I*. As climate and the other topographic variables had a strong spatial structure, we also checked for spatial autocorrelation in the residuals of the model [49], [50].

All statistical analyses were carried out with the R 2.15.3 programme [51] (see S1 Dataset).

## Result and Discussion

### Influence of site

Mean values and pooled standard deviations of the stable isotope ratios were computed for each site: Table 1 summarises these results (detailed data are provided in S1 Table). Comparison between the pooled and experimental SDs showed intra-site variability to be high for δ^13^C and δ^15^N (pooled SD 4.5 times greater than analytical precision for δ^13^C and 3 times greater for δ^15^N) and low for δ^2^H and δ^18^O (pooled SD 1.5 times greater than analytical precision for δ^2^H and 1.25 times greater for δ^18^O).

There were no significant differences in δ^13^C among sites (analysis of variance, *F* = 1.7, *p* = 0.06), the values ranging between-27.1 ‰ and-29.0 ‰, although there was considerable within-site variation. These data confirm a very weak site effect on δ^13^C values [27]. Several studies have reported that ^13^C fractionation is strictly dependent on inherent physiological tree response (e.g. stomatal conductance and photosynthetic rate) to external factors. In accordance with carbon isotope theory [52], our results indicate that individual-specific variability may be more important than local environmental factors. In light of these results, δ^13^C was excluded from the subsequent analysis.

On the other hand, the ANOVA showed that the δ^18^O, δ^2^H and δ^15^N values differed significantly (*p* < 0.001) among sites. These results are consistent with the strong influence of site on δ^18^O, δ^2^H and δ^15^N reported by other researchers [10], [28], [53], [54].

### Explanatory variables

The relationship between the stable isotope ratios in needles and the explanatory variables was tested by means of the Pearson’s correlation coefficient. Table 2 reports these results. Accepting only a significance level of *p* <0.05 we did not find any significant relationships between isotope ratios (*p* <0.05), suggesting that, although stable isotope ratios are controlled by the same environmental factors, they appear to be relatively independent in needles and likely reflect the effect of different environmental proxies.

**Table 2:** Coefficients of correlation between isotope ratios and spatial/environmental variables.

	Mean annual temperature(°C)	Mean summer temperature(°C)	Autumn precipitation(mm)	Altitude(m)	Global irradiance(W/m^2^)	Topographic Wetness Index (TWI)	Distance from the coast(km)	Distance from motorway	Longitude(degrees/minutes/seconds)	Latitude(degrees/minutes/seconds)
δ^18^O		0.58**							-0.68***	-0.60**
δ^2^H			-0.67**		0.50*		-0.71***			
δ^15^N								0.58**		

* *p* < 0.05;

**p < 0.01;

*** *p* < 0.001

Values not significant at *p* < 0.05 are not reported.

### Climate and geographical effects

Variability in δ^2^H values is known to be controlled by the same factors influencing δ^2^O values [22], [55], which are mainly determined by the isotopic composition of the soil water and by leaf water. Nevertheless, it appears that here other fractionation effects are involved. In a previous study [36] we reported no relationship between δ^2^H and δ^2^O values in Norway spruce. Our results confirm δ^2^H and δ^2^O values to be affected by different climatic factors, since δ^2^H are strongly correlated with autumn precipitation while δ^2^O correlates only with mean annual temperature.

The strongest correlation was obtained for distance from the coast as a predictor for δ^2^H values (p < 0.001), which also negatively correlated with autumn precipitation (*p* < 0.01) and were also positively influenced by global irradiance (*p* < 0.05). The δ^18^O value correlated positively with mean summer temperature (p < 0.01) and negatively with longitude (*p* < 0.001) and latitude (*p* < 0.005). A depletion in ^2^H of precipitation is expected with increasing distance from the coast to the inner continent. This process is referred to as the “*continental effect*” and is known to contribute to heavy isotope depletion at a rate of around-3‰ per 100 km for ^2^H [45]. Furthermore, as precipitating air masses travel from inter-tropical regions toward the poles gradual depletion in ^2^H and ^18^O occurs. This phenomenon, known as “*latitude effect*”, goes a long way to explaining the negative relationship between δ^18^O values and latitude. We do not have a clear explanation for the longitude effect but we hypothesise that the negative relationship between δ^18^O values and longitude may be due to two interacting effects: a) the “*continental effect*” due to the geographical position of the sampling sites (many sites in the inner part of the Alps are also at high longitudes, Fig. 1); b) the presence of a longitudinal precipitation gradient from east to west in the European Alps. Differences along longitude have also been observed in ^2^H of bulk Italian olive oils between the Adriatic and Tyrrhenian coasts as a consequence of the different sources and isotopic compositions of rainfall as well as the different climatic conditions on the two coasts [56]. These results are consistent with previous studies [23], [43], [45], and confirm that δ^2^H and δ^18^O values strongly reflect the isotope signal of precipitating air masses. The clear, stable relationship between isotope values and climate parameters confirms a weak influence of plant-individual variability on isotope fractionation during photosynthesis. In agreement with Araguás-Araguás [43], we confirm that both δ^18^O and δ^2^H vary in a predictable manner and strongly correlate with environmental variables.

Contrary to expectations, we did not find either δ^18^O or δ^2^H correlated with altitude, probably because we considered a wide area characterised by fairly significant climatic variability which may have masked the altitude effect.

### Effects of nitrogen dioxide

We have found evidence that the ^15^N signal from NO_2_ anthropogenic emissions persists in spruce needles. Indeed, the positive correlation (p<0.01) between distance from motorway and δ^15^N in spruce needles (Table 2) undoubtedly reflects enrichment by heavy ^15^NO_2_ caused by car emissions; δ^15^N of NO_2_ due to anthropogenic emissions differs significantly from the natural background δ^15^N in the soil [57]. Enriched ^15^N in NO_2_ pollution is reported in the literature, and since trees may take up N from NO_2_ this isotopic signal is reflected in plant tissue. For instance, Amman et al.[25] reported higher ^15^N of NO and NO_2_ in spruce needles from sites close to car exhaust emissions. Saurer et al. [26] found that the δ^15^N signal was clearly related to distance from motorway, and reported that the pollutant signal was evident in direct proximity to the motorway but disappeared at a distance of 150 m due to strong dilution of pollution plumes [58]. Our findings confirm these findings as there were no further significant relationships between δ^15^N values in spruce needles and distance from motorway at sites more than 150 m away from the motorway (i.e., sites 1, 6, 9, 10, 12, 14 and 16).

Contrary to what is reported in the literature, we didn’t find correlation between NO_2_ concentrations and δ ^15^N values in spruce needles. One explanation could be the coarse resolution of the NO_2_ concentration map that we used (10.000 meters). Moreover, this map fails to take into account nitrogen emission from different sources. Indeed, agricultural activity and combustion from fossil fuels have been shown to give rise to large variability in the ^15^N/^14^N. It should be born in mind that anthropogenic fractionation of N isotopes varies according to the heat of combustion. Therefore, further analysis focussing on this relationship should take into account specific atmospheric nitrogen pollution.

### Multivariate models and spatial autocorrelation

**Fig 2:**
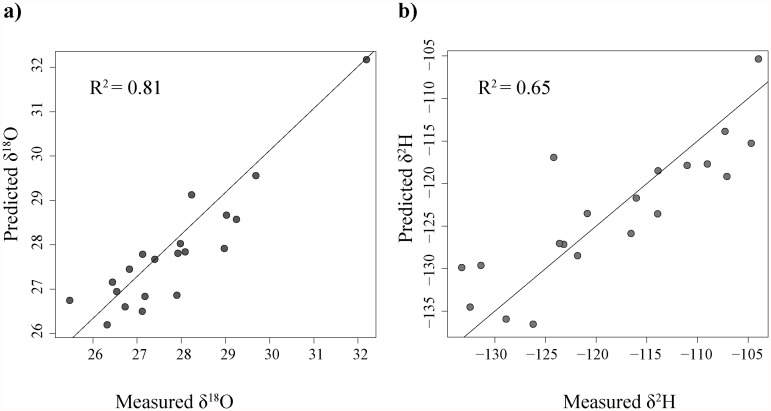
Scatter graphs of the predicted stable isotope values *vs* measured stable isotope values for the two elements: oxygen (a); deuterium (b). Adjusted R^2^ is reported for each plot.

A multiple linear regression analysis was conducted to improve the common signal of explanatory variables. The stepwise procedure performed on eleven explanatory variables (latitude, longitude, mean annual and mean summer temperatures, autumn precipitation, altitude, global irradiance, TWI and distance from the coast) selected significant predictors for δ^18^O and δ^2^H (Table 3). No predictive model could be established at *p* < 0.05 for δ^15^N. Each final model was assessed following all the normal procedures, including normality and homoscedasticity of the residuals and leverages; assumptions of linear regression were met in all cases. Scatter graphs (Fig. 2) show the relationships between predicted and measured isotope values for the two proxies. In agreement with a previous study [36], temperature is expected to positively influence ^18^O and ^2^H values. Indeed, the best predictive model was found using mean summer temperature and altitude as explanatory variables for δ^18^O values (adjusted *R* ^2^, 0.81; *p* < 0.001). Furthermore, these findings support the results reported by Holdsworth et al. [44], who demonstrated that depletion in ^18^O occurs with increasing elevation (-0.15‰ to-0.50‰ per 100m height). Prediction of δ^2^H values was significantly related to mean summer temperature, distance from the coast and TWI, although to a lesser extent; the final model accounted for 65% of the total variation.

**Table 3:** Summary of multiple regression models for δ^18^O, δ^2^H and δ^13^C.

	Selected environmental variables	*p*-value	Adjusted *R* ^2^ of the model
δ^18^O	Mean summer temperature	< 0.001	0.81 ***
	Altitude	< 0.001	
δ^2^H	Mean summer temperature	0.05	0.65 ***
	Distance from the coast	0.01	
	Global irradiance	0.09	
	TWI	0.05	

* *p* < 0.05;

**p < 0.01;

*** *p* < 0.001

Variable selection was made by *F*-test.

### Geostatistical analysis

The above correlations do not take into account the geographical location of the site. We therefore calculated Moran’s Index on all the isotope ratios to investigate their spatial patterning. We found positive spatial autocorrelations for δ ^18^O (*I* = 0.4; p < 0.05) and δ ^2^H (*I* = 0.6; p < 0.001), but no significant spatial autocorrelations for δ^13^C and δ^15^N and none at all in the residuals of OLS regression. The spatial structures of δ ^18^O and δ ^2^H in Norway spruce needles probably reflects the spatial structure of the meteoric water isotopic compositions [59], [60]. As several researchers have reported [23], [45], [54], [61], [62], spatial variability in δ ^18^O and δ ^2^H of precipitation reflects a combination of rainout effects and the effect of recycling on air masses. The absence of spatial autocorrelation in δ^15^N is due to the fact that δ^15^N values in needles are expected to reflect local emission of anthropogenic nitrogen compounds [53] and soil isotope composition and may therefore greatly vary even between closely neighbouring sites. Therefore, in order to verify spatial structure in δ ^15^N values, future studies should employ a smaller geographic scale and consider a greater number of sample sites.

Overall, these results are in line with other studies and show that δ^18^O and δ^2^H have a strong spatial structure and are primarily influenced by regional and local-scale processes and less so by individual-specific variations. Since these proxies don’t significantly correlate with each other, we believe that dendroprovenancing studies must take both δ ^18^O and δ^2^H values into account.

## Conclusions

We found that δ ^18^O and δ^2^H values were the best proxies in dendroprovenancing; in contrast to the conclusions reached by Kagawa and Leavitt [10] for the south-western United States, δ^13^C did not significantly differ among sites and may therefore not be a suitable proxy to pinpoint the geographic origin of trees in the Alps. This discrepancy may be due either to different geographic scales (ours was smaller, within a range of 600 km) or different plant materials.

Although we found that δ^15^N values were highly site-dependent, our model showed only weak predictability for the variability of this proxy. Since δ^15^N values are mainly influenced by anthropogenic emissions on a local scale [53], we urge further studies on a smaller geographic scale. Furthermore, we must take into account that some countries may use N as a fertilizer [12], rendering dendroprovenancing problematic and difficult to interpret.

On the basis of these findings, we consider spatial distribution maps, at least for δ ^18^O and δ^2^H, to be possible even on a limited scale (such as the alpine area). Although the OLS regression was highly significant, we believe that model quality can be further improved, at least for δ ^18^O and δ^2^H. It should be borne in mind that an OLS regression only uses data available at the target location and may fail to take into account existing spatial autocorrelations [49]. Indeed, these isotope ratios displayed high spatial structures correlating with several environmental features, therefore a more appropriate method might be one that takes the covariance between spatial variables into account. For these reasons, we believe future research should focus on methods that take into account spatial interpolation. Co-kriging may be a reliable method as it simultaneously accounts for spatial autocorrelation of proxies and computes the cross-correlations between these variables [63]. Moreover, this geostatistical method also provides an estimated standard error map. Given that we sampled 20 sites with a spatial range of about 600 km, spatial interpolation may lead to biased results [49] and for this reason our work here should be regarded as a pilot study. We therefore suggest increasing the number of sample sites in order to perform proper spatial interpolation.

As a general conclusion, our study demonstrates the potential of stable isotope measurements in identifying the geographic origin of trees, and, although further studies are clearly required, we believe this to be a promising method for verifying the origin of wood.

## Supporting Information

S1 Dataset R script used in this study. (TXT)

S1 Table Sampled sites and related stable isotope values. (PDF)
