# Supplementary material for: Oxygen and Hydrogen Stable Isotope Ratios of Bulk Needles Reveal the Geographic Origin of Norway Spruce in the European Alps
Source: PLoS One. 2015 Mar 5;10(3):e0118941. doi: 10.1371/journal.pone.0118941 (PMC4351073; doi:10.1371/journal.pone.0118941)
Supplement: S1 Table — (PDF) [file pone.0118941.s002.pdf]

number	sites	$\delta^{18}O$	$\delta^{2}H$	$\delta^{13}C$	$\delta^{15}N$
1	Saint-ch	32	-106	-27.8	-0.6
1	Saint-ch	31.1	-110	-28.6	-0.6
1	Saint-ch	33.2	-99	-29.3	-1.4
1	Saint-ch	32	-107	-27.5	-0.2
1	Saint-ch	32.3	-104	-29.3	-1.1
2	Villar	27.9	-139	-28.1	-5.2
2	Villar	30.1	-135	-27.6	-4.3
2	Villar	29	-133	-26.5	-0.7
2	Villar	30.2	-111	-29.1	-4.1
2	Villar	28.7	-133	-27.4	-3.9
3	cogne	27.8	-142	-28	-5
3	cogne	28.4	-144	-27.7	-5.3
3	cogne	28.1	-134	-26.7	-4.2
3	cogne	28.6	-134	-29	-4.9
3	cogne	28.1	-142	-28.3	-4.7
4	ayes	28	-129	-27.3	-3.6
4	ayes	28.1	-125	-27.2	-3.5
4	ayes	28	-133	-26.3	-3.4
4	ayes	27.8	-128	-27.6	-3.9
4	ayes	27.8	-131	-27.3	-3.8
5	medel	27.1	-131	-26	-2.6
5	medel	27	-130	-28.2	-3
5	medel	26.6	-133	-28	-4.3
5	medel	26.4	-133	-28.2	-4.8
5	medel	26.3	-137	-27.5	-4.4
6	mezzoldo	29.7	-118	-28.9	1.1
6	mezzoldo	29.9	-111	-28.9	0.9
6	mezzoldo	29.8	-114	-28.6	1
6	mezzoldo	29.4	-117	-28.4	1
6	mezzoldo	29.3	-125	-27.5	0.9
7	savognin	26.6	-153	-25.4	-1.7
7	savognin	27.7	-142	-26.7	0.2
7	savognin	27.7	-134	-29.9	-0.7
7	savognin	27.5	-129	-29.6	-0.8
7	savognin	27.3	-145	-29	-1.7
8	arnoga	28.1	-122	-27.7	-6.8
8	arnoga	27.2	-122	-27.3	-6.1
8	arnoga	28.6	-115	-26.5	-6.5
8	arnoga	28.3	-123	-27.9	-6.5
8	arnoga	28	-119	-28.3	-5.1
9	pfunds	29.4	-141	-27.5	-4.1
9	pfunds	28.5	-150	-27.2	-4.8
9	pfunds	28.4	-133	-28.7	-4.6
9	pfunds	28.7	-145	-27.1	-4.5
9	pfunds	29.6	-139	-27.4	-4.5
10	pellizzano	27.2	-130	-28.5	-2.2
10	pellizzano	27.9	-128	-28.5	-2.6
10	pellizzano	27.8	-120	-28.5	-2.4

10	pellizzano	28	-127	-29.1	-2.3
10	pellizzano	28.4	-131	-28.9	-2.9
11	martell	28.9	-115	-28.1	2.4
11	martell	27.1	-141	-27.7	-2.2
11	martell	26.8	-130	-27.6	-3.2
11	martell	28.5	-119	-27.2	-2.7
11	martell	27.9	-139	-28.4	-1.7
12	sautens	27.1	-135	-27.4	-2.1
12	sautens	26.2	-137	-27.9	-2.1
12	sautens	26.9	-132	-30	-2.5
12	sautens	27.1	-133	-28.1	-1.8
12	sautens	26.6	-143	-28.3	-1.9
13	caldonazzo	29.4	-102	-27.9	-6.2
13	caldonazzo	29.9	-107	-27.1	-6.5
13	caldonazzo	27.1	-111	-30.3	-7.7
13	caldonazzo	29.1	-112	-29.5	-8.2
13	caldonazzo	29.4	-110	-30	-8.5
14	primiero	27	-110	-28.4	0
14	primiero	27	-117	-28.1	-0.1
14	primiero	27	-128	-26.9	-0.8
14	primiero	27.1	-116	-27.8	-0.1
14	primiero	27.3	-97	-29.3	-1.7
15	agordo	25.5	-115	-28.5	-1.3
15	agordo	25.4	-112	-28.4	-1.3
15	agordo	25.1	-112	-28	-1.4
15	agordo	26	-95	-29.7	-1.3
15	agordo	25	-121	-27.7	-1
16	cortina	26.7	-121	-27.3	0.1
16	cortina	26.8	-123	-27.3	0.1
16	cortina	27.2	-123	-27.4	0.5
16	cortina	26.8	-121	-27.1	-0.2
16	cortina	27.8	-111	-28.4	0.6
17	erto	27	-97	-29.4	-3.9
17	erto	25.8	-85	-32.4	-2.5
17	erto	27.5	-104	-27.7	-2.5
17	erto	28.1	-107	-28.5	-3.9
17	erto	27.2	-129	-27.7	-2.5
18	cadore	26.5	-113	-29	-5.4
18	cadore	26.8	-120	-29.4	-5.5
18	cadore	26.6	-118	-26.3	-6.2
18	cadore	26.2	-116	-25.9	-6
18	cadore	26.3	-118	-26	-6.1
19	barcis	26.5	-102	-26.2	-2.1
19	barcis	25.8	-112	-26.5	-1.9
19	barcis	25.6	-115	-27.5	-2.6
19	barcis	26.1	-112	-27.6	-2.4
19	barcis	27.3	-102	-27.5	-1.8
20	ugovizza	25.4	-127	-28	-7.2
20	ugovizza	26.5	-125	-28.4	-6.9
20	ugovizza	27.3	-127	-28.7	-7.2

20	ugovizza	26.1	-130	-27.6	-7.5
20	ugovizza	26.5	-120	-28.9	-7.3
